# A FtsH-Like Zinc Metalloprotease, OsFtsH1, Coordinates Chloroplast Development and Photosystem II Homeostasis in Rice

**DOI:** 10.1186/s12284-026-00936-2

**Published:** 2026-07-11

**Authors:** Kangwei Liu, Qiao Yu, Haiyang Feng, Mengna Wang, Qiang Dai, Jiayi Chang, Chao Zhang, Hengxiu Yu

**Affiliations:** 1 Jiangsu Key Laboratory of Crop Genomics and Molecular Breeding, Zhongshan Biological Breeding Laboratory, Key Laboratory of Plant Functional Genomics of the Ministry of Education, Jiangsu Co-Innovation Center for Modern Production Technology of Grain Crops, Agricultural College of Yangzhou University, Yangzhou, China; 2 Yangzhou Modern Seed Innovation Institute, Yangzhou, 225600 China

## Abstract

Chloroplast development is a fundamental process underlying photosynthesis and plant growth, yet its molecular regulatory mechanisms remain to be fully elucidated. In this study, we identified an albino rice mutant, which exhibits drastically reduced chlorophyll content and defective chloroplast ultrastructure. Through map-based cloning, coupled with CRISPR/Cas9-mediated gene editing and complementation assays, we verified that the target gene encodes a zinc metalloprotease of the FtsH protein family, OsFtsH1. The expression of *OsFtsH1* is light-inducible, and its encoded protein localizes specifically to chloroplasts. RNA-sequencing (RNA-seq) analysis revealed broad differential expression of photosynthesis-related genes in *osftsh1* mutants. qRT-PCR further demonstrated that plastid-encoded genes involved in chloroplast biogenesis are markedly downregulated at the transcriptional level. Functional assays indicated that OsFtsH1 sustains the homeostasis of D1, D2 and CP43, core proteins of the photosystem II (PSII), and collaboratively regulates chloroplast development through interactions with three key proteins: the chloroplast signaling protein OsCPL1, the PSII oxygen-evolving complex component OsPsbO, and the photosynthetic electron transport protein OsFd1. Additionally, the expression of *OsFtsH1* is induced by indole-3-acetic acid (IAA) and abscisic acid (ABA), and its overexpression markedly enhances rice sensitivity to these two phytohormones. Collectively, our findings unravel the multifaceted and crucial functions of *OsFtsH1* in orchestrating chloroplast development, photosynthetic machinery homeostasis, and stress response in rice.

**Supplementary Information:**

The online version contains supplementary material available at https://doi.org/10.1186/s12284-026-00936-2.

## Introduction

Chloroplasts are the core organelles for photosynthesis in plants. As unique organelles, they capture light energy and convert it into chemical energy through processes such as CO_2_ fixation and carbon skeleton biosynthesis (Sakamoto et al. 2003; Jarvis and López-Juez 2013). They are also responsible for synthesizing various metabolites, including tetrapyrroles, amino acids, lipids, terpenoids, and hormones (Li et al. 2018). As semiautonomous organelles, chloroplasts possess their own genome and protein synthesis machinery; however, they can only synthesize a subset of their proteins. Most chloroplast proteins are synthesized on cytoplasmic ribosomes and subsequently imported into the organelles (Wu et al. 2016). Chloroplast development is a sophisticated process involving differentiation from proplastids into mature chloroplasts, requiring coordinated regulation by plastid and nuclear genes through "plastid signaling". This process specifically consists of four stages: Stage I (leaf base meristem stage) involves the activation of plastid DNA synthesis and replication to maintain plastid number and DNA content in dividing cells; Stage II (cell expansion stage) is characterized by a significant increase in plastid transcriptional activity and RNA levels. During this stage, plastid genes encoding the transcription/translation machinery components (e.g., *rpoB*-*rpoC1*-*rpoC2*, *rps16*, and rRNA genes) are preferentially transcribed by the nuclear-encoded plastid RNA polymerase (NEP), laying the foundation for photosynthetic system assembly (Hajdukiewicz et al. 1997); Stage III (photosynthetic system assembly stage) involves the large-scale expression of plastid genes encoding photosynthetic apparatus components (e.g., *psaA*-*psaB*, *psbA*) as well as their nuclear counterparts.

At this stage, transcription of plastid genes shifts to being primarily driven by the plastid-encoded RNA polymerase (PEP). Activation of nuclear gene expression by "plastid signaling" is essential, and its disruption leads to impaired chloroplast development (De Santis-MacIossek et al. 1999); Stage IV (maturation and maintenance stage) is marked by an overall decrease in chloroplast transcriptional rates. However, continuous synthesis of the labile PSII reaction center proteins D1 and D2 is maintained through light-induced differential transcription of *psbA* and *psbD*-*psbC*, ensuring the stability of photosynthetic function. To date, the molecular regulatory mechanisms governing chloroplast development in higher plants remain largely elusive (Pfalz and Pfannschmidt 2013).

Filamentous temperature-sensitive H (FtsH) proteases are a family of ATP-dependent zinc metalloproteases belonging to the AAA⁺ (ATPase associated with diverse cellular activities) protease subfamily (Ito and Akiyama 2005) and are involved in various cellular processes. Uniquely membrane-anchored, FtsH proteases are widely distributed in prokaryotes as well as in the mitochondria and chloroplasts of eukaryotes (Akiyama et al. 1995; Janska et al. 2013). The ATPase domain of FtsH possesses unfoldase activity, transporting substrates through a narrow pore into the proteolytic chamber to mediate the degradation of integral membrane proteins. Substrate recognition by FtsH requires at least 20 amino acid residues at either the N- or C-terminus of the target protein (Chiba et al. 2000, 2002). Structurally, thylakoid-localized FtsH proteases contain an N-terminal transmembrane domain, with their C-termini extending into the stroma and housing the ATPase and protease domains (Lindahl et al. 1996). In the thylakoid membranes of Arabidopsis, FtsH proteases assemble into heterohexameric complexes consisting of two subunit types: Type A (FtsH1, FtsH5) and Type B (FtsH2, FtsH8). Among these, FtsH2 is the most abundant, followed by FtsH5, FtsH8, and FtsH1 (Sakamoto et al. 2003; Yu et al. 2004, 2005). As core regulators of chloroplast protein homeostasis, FtsH proteases not only participate in thylakoid membrane biogenesis and maintenance but also act as key mediators in the degradation of photodamaged D1 protein during the PSII repair cycle. The D1 protein, a core component of the PSII reaction center, is the primary target of photodamage under light stress; failure to promptly degrade D1 proteins with altered conformations caused by excessive light energy absorption will compromise PSII. Additionally, FtsH proteases are involved in the singlet oxygen (^1^O_2_)- and EXECUTER1 (EX1)-dependent retrograde signaling pathway. Dysfunction of FtsH proteases leads to drastically enhanced sensitivity to high-light stress accompanied by severe photoinhibition (Bailey et al. 2002; Murata et al. 2007; Kato et al. 2009, 2012; Nixon et al. 2010; Wang et al. 2016). Furthermore, FtsH proteases are highly conserved across photosynthetic organisms including cyanobacteria and tobacco, and their gene duplication events are considered to be closely related to the evolution of oxygenic photosynthesis (Komenda et al. 2007; Boehm et al. 2012; Kato et al. 2012).

The Arabidopsis genome encodes 12 FtsH proteins, nine of which (FtsH1, 2, 5, 6, 7, 8, 9, 11, and 12) localize to chloroplasts, while the remaining three (FtsH3, 4, and 10) are targeted to mitochondria. FtsH isoforms exhibit marked specificity in subcellular localization, complex assembly, and physiological functions (Sakamoto et al. 2003; Urantowka et al. 2005). Within chloroplasts, type A and type B subunits are incorporated into thylakoid membranes via distinct pathways: FtsH5 (type A) depends on the Sec translocation system, whereas FtsH2 (type B) relies on the Tat transport pathway. The synergistic interaction between these two subunit types is essential for the assembly of functionally active heterohexameric complexes (Yu et al. 2004, 2005; Zaltsman et al. 2005b; Rodrigues et al. 2011). Mutant analysis shows that loss of FtsH2 function (*var2* mutant) causes severe leaf variegation, while deficiency in FtsH5 (*var1* mutant) results in a milder leaf variegation. Single mutations in FtsH1 or FtsH8 do not cause obvious visible defects; however, double mutants such as *ftsh1 ftsh5* and *ftsh2 ftsh8* exhibit an albino-like phenotype, indicating that these subunits play indispensable and partially redundant roles in chloroplast differentiation (Chen et al. 2000; Takechi et al. 2000; Sakamoto et al. 2002; Yu et al. 2005; Zaltsman et al. 2005b). Beyond its central role in PSII repair, FtsH is also involved in the degradation and assembly of the cytochrome b₆f complex and Photosystem I (PSI) complex, as well as in the turnover of light-harvesting chlorophyll a/b-binding proteins associated with PSI. Impairment of FtsH function thus compromises the integrity of the photosynthetic electron transport chain (Ostersetzer and Adam 1997; Malnoë et al. 2014; Wei et al. 2014; Järvi et al. 2016; Bujaldon et al. 2017). Regarding regulatory mechanisms, FtsH protease activity is modulated by its dynamic oligomerization capability, post-translational modifications (e.g., phosphorylation, disulfide bond formation), and its own rapid turnover. Under high-light stress, *FtsH* gene expression is upregulated and protein degradation is accelerated, enabling plants to adapt to environmental stress and maintain functional stability (Zaltsman et al. 2005a; Reiland et al. 2009; Stael et al. 2012; Li et al. 2017; Wang et al. 2017). Although the physiological functions of FtsH in photosynthetic organisms have been widely documented, the precise molecular mechanisms underlying substrate-specific recognition, retrograde signaling, and the crosstalk among different regulatory pathways remain to be fully elucidated.

The rice genome is currently known to encode 9 FtsH proteins (Yu et al. 2005; Zhang and Sun 2009). Among them, OsFtsH3, OsFtsH4, and OsFtsH5 are predicted to localize to mitochondria, while the rest are targeted to chloroplasts (Zhang and Sun 2009). Previous studies have revealed that knockout of the *FtsH2* gene leads to an albino seedling phenotype in rice (Wu et al. 2021). However, research into the functions and molecular mechanisms of the FtsH family genes in rice remains limited. In this study, we cloned the *OsFtsH1* gene using an albino mutant, and validated its pivotal role in sustaining normal chloroplast development. Its core functions include regulating the expression of chloroplast-related genes, modulating D1, D2 and CP43 protein abundances, and coordinating chloroplast development through interaction with OsCPL1, OsPsbO and OsFd1. Furthermore, we found that elevated expression of *OsFtsH1* enhances the sensitivity of rice seedlings to indole-3-acetic acid (IAA) and abscisic acid (ABA). These findings clarify the function of OsFtsH1 in chloroplast development and the maintenance of photosynthetic system homeostasis, and provide novel insights into the molecular regulatory network governing chloroplast development in rice.

## Results

### Isolation and Phenotypic Description of *the White Leaf Lethal Seedling1* (*wlls1*) Mutant

**Fig. 1:**
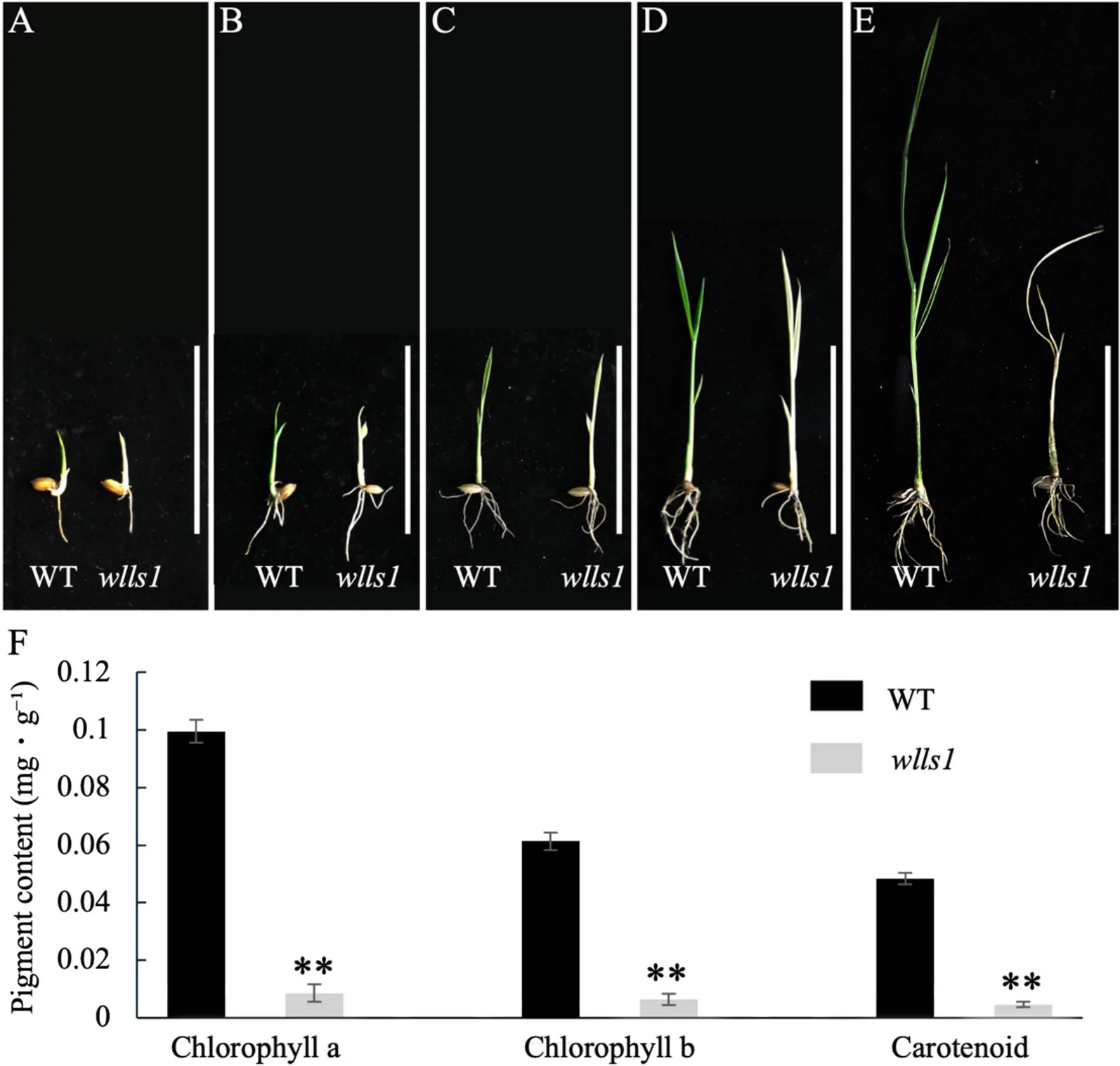
Phenotypic characterization of the *wlls1* mutant. **A**–**E** Phenotypic comparison of wild-type (WT) and *wlls1* seedlings at 2, 5, 8, 11 and 14 days after germination. Bars = 5 cm. **F** Photosynthetic pigment contents in 10-day-old WT and *wlls1* seedlings. Data are presented as mean ± standard deviation (SD) (n = 3). Asterisks indicate significant differences based on a two-tailed Student’s t test (**P < 0.01).

A rice mutant exhibiting an albino phenotype was isolated from a mutant library constructed via ^60^Co γ-ray irradiation. This mutant, designated as *white leaf lethal seedling1* (*wlls1*), exhibited fully white leaves from the first leaf (Fig. 1A, B). The albino leaf phenotype persisted throughout the entire growth period, and the mutant seedlings gradually withered and eventually died by the fourth-leaf stage (Fig. 1C–E). This albino-lethal trait was stably inherited across successive generations.

The albino phenotype in rice seedlings is frequently associated with deficiencies in photosynthetic pigment biosynthesis. To investigate this in *wlls1*, we quantified the major photosynthetic pigments in both wild-type (WT) and mutant seedlings at the three-leaf stage. Comparative analysis revealed a drastic reduction in the content of all pigments measured. Specifically, the contents of chlorophyll a (WT: 1.0873 ± 0.0123 mg/g; *wlls1*: 0.0772 ± 0.0059 mg/g), chlorophyll b (WT: 0.3663 ± 0.0058 mg/g; *wlls1*: 0.1083 ± 0.0098 mg/g), and carotenoids (WT: 0.3142 ± 0.0047 mg/g; *wlls1*: 0.0413 ± 0.0018 mg/g) were significantly lower in the *wlls1* mutant compared to the WT controls (Fig. 1F).

### Chloroplast Development is Impaired in *wlls1*

**Fig. 2:**
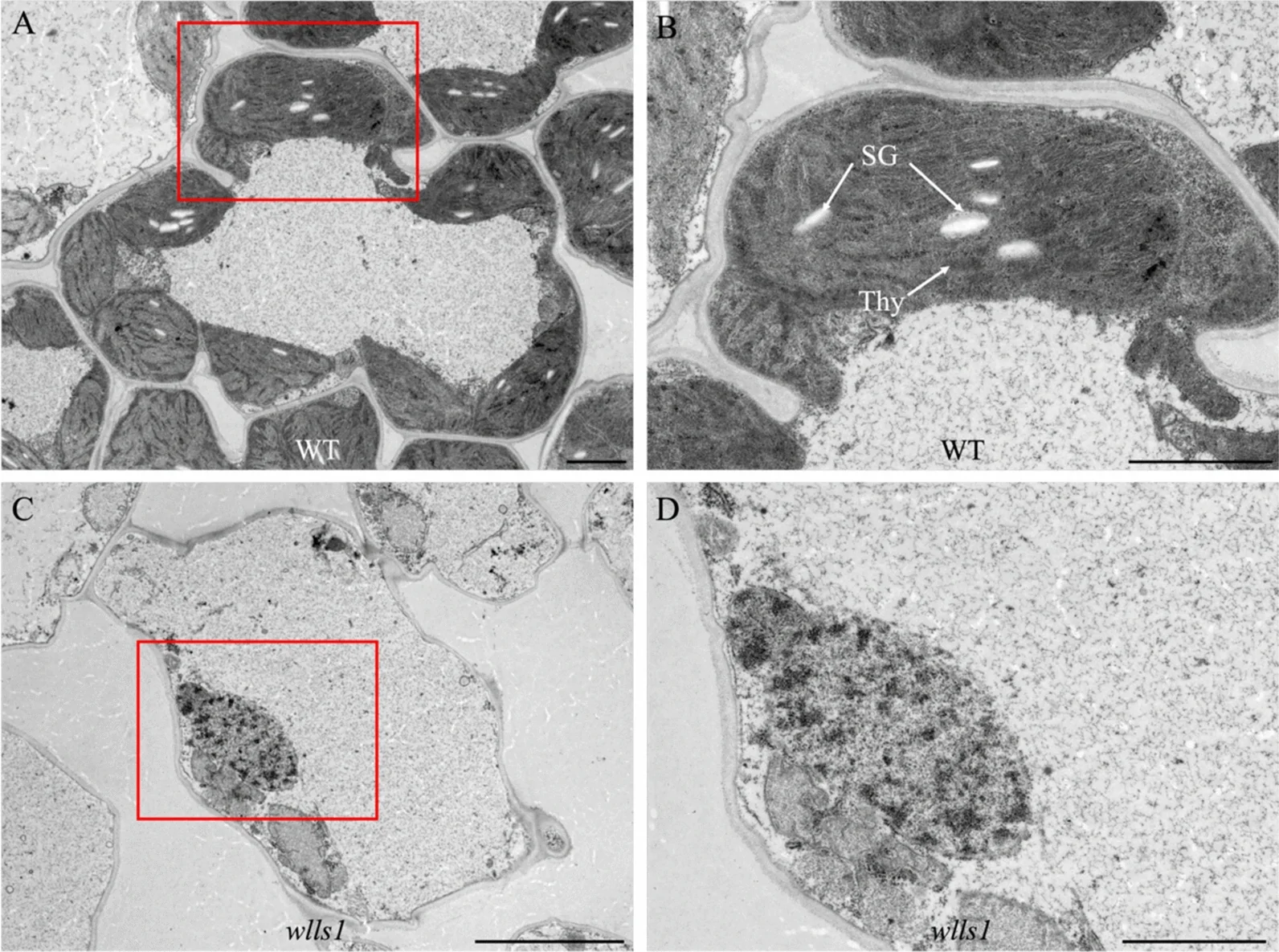
Transmission electron micrographs of leaf tissues from WT and *wlls1* seedlings. **A** WT chloroplast at low magnification; the red box indicates the region shown in (**B**). **B** Higher-magnification view of the region outlined in red in (**A**), showing starch granules (SG) and thylakoids (Thy). **C** *wlls1* mutant chloroplast at low magnification; the red box indicates the region shown in (**D**). **D** Higher-magnification view of the region outlined in red in (**C**). Fully expanded second true leaves were collected from plants at the three-leaf stage. Bars = 1 μm

Chloroplast biogenesis relies on sustained chlorophyll biosynthesis and the subsequent assembly of photosynthetic apparatus (Wang and Grimm 2015). The albino leaf phenotype of the *wlls1* mutant indicated impaired chloroplast development. To verify this hypothesis, we compared the chloroplast ultrastructure in wild-type (WT) and *wlls1* leaves using transmission electron microscopy (TEM). Mesophyll cells of the WT contained numerous well-differentiated chloroplasts with highly stacked thylakoid membranes and distinct starch granules (Fig. 2A, B). In contrast, mesophyll cells of the *wlls1* mutant exhibited a dramatic reduction in chloroplast abundance. The few residual chloroplasts were poorly developed, lacked organized grana stacks, and instead contained irregular electron-dense aggregate structures indicative of the aberrant accumulation of photosynthetic membrane components and protein complexes (Fig. 2C, D). Collectively, these TEM results confirm that the *wlls1* mutation disrupts the normal progression of chloroplast development.

### Map-Based Cloning of the Causal Gene *WLLS1*

To clone the target gene, we first performed genetic analysis. In the segregating population, the phenotypic segregation ratio of WT to mutant plants was 127:46, consistent with a typical 3:1 Mendelian segregation pattern (χ^2^_(1)_ = 0.23, P > 0.05). This result indicates that the mutant phenotype is governed by a single recessive nuclear gene. For identifying the causal gene, we adopted a map-based cloning approach. Preliminary linkage analysis anchored the target locus to a 2.38 Mb genomic interval on the long arm of chromosome 6. Subsequent fine mapping narrowed down the candidate region to a critical 230 kb segment. According to the Rice Genome Annotation Project, this region contains 40 candidate genes. Sequencing analysis identified a 4-bp deletion in the first exon of LOC_Os06g51029 (corresponding to *OsFtsH1*) in the *wlls1* mutant, which is predicted to cause a frameshift mutation in the encoded protein (Figs. S1A, S1B and S2).

**Fig. 3:**
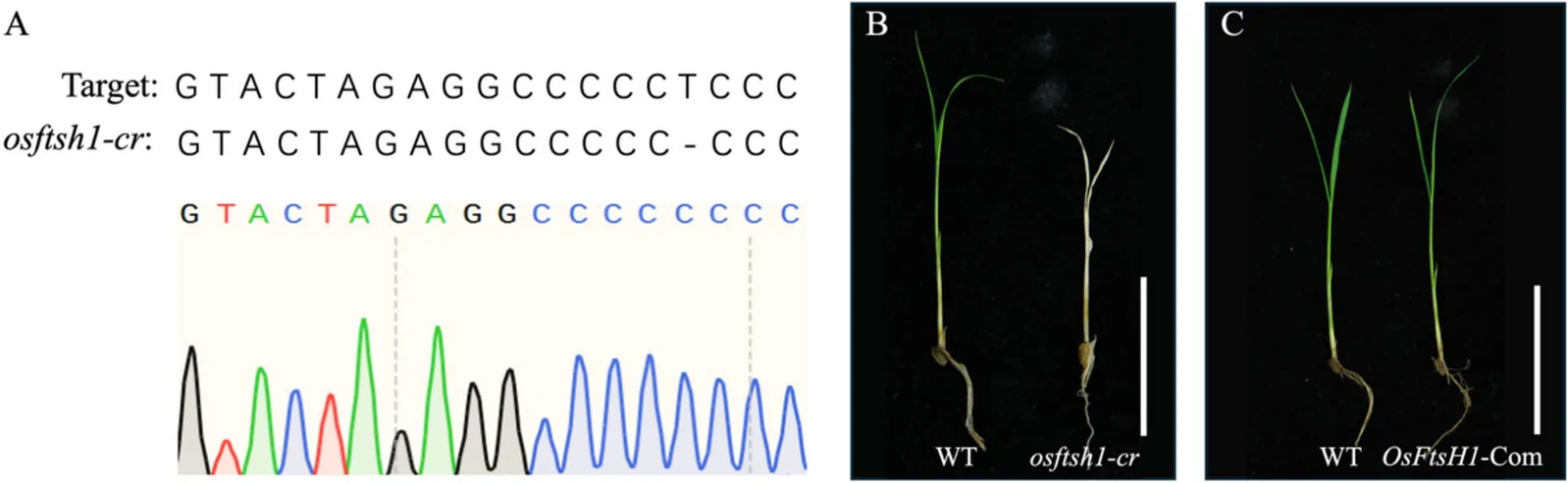
Characterization and functional verification of *OsFtsH1.* **A** Target sequence for CRISPR/Cas9-mediated editing of *OsFtsH1* and genotyping results of the resulting knockout mutant *osftsh1-cr*. **B** Phenotypes of *osftsh1-cr* seedlings at 12 days after germination. **C** Phenotypes of complemented *OsFtsH1*-Com seedlings at 12 days after germination. Bars = 5 cm

To functionally validate *OsFtsH1*, we performed a CRISPR/Cas9-mediated gene knockout in the Y8 background, targeting a site in the first exon of *OsFtsH1* to fully disrupt its function. Among the edited lines, we obtained a mutant, designated *osftsh1-cr*, harboring a 1-bp deletion predicted to cause a frameshift and premature translation termination (Fig. 3A, S2). The *osftsh1-cr* mutants displayed a white-leaf phenotype followed by seedling lethality, phenotypically identical to the original *wlls1* mutant (Fig. 3B). We further quantified *OsFtsH1* transcript levels in *wlls1* and *osftsh1-cr* seedlings via qRT-PCR. The transcript abundance of *OsFtsH1* was significantly reduced in both mutants relative to wild-type plants, confirming that nonsense-mediated mRNA decay (NMD) is triggered in both mutant alleles and diminishes the accumulation of functional *OsFtsH1* transcripts (Fig. S3).

For complementation assays, the full-length *OsFtsH1* cDNA under the control of the *ACTIN* (*ACT*) promoter was introduced into *wlls1* calli via *Agrobacterium tumefaciens*-mediated transformation. From the progeny of the transformants, homozygous complemented T_2_ plants were obtained and subjected to phenotypic analysis. The albino phenotype of the *wlls1* mutant was fully restored to the WT state in these complementation lines (Fig. 3C). Collectively, these results confirm that the loss of function of *OsFtsH1* is responsible for the albino seedling phenotype in the *wlls1* mutant.

### *OsFtsH1* Encodes a Zinc Metalloprotease

OsFtsH1 is a member of the evolutionarily conserved plant FtsH family of ATP-dependent zinc metalloproteases. This protein harbors conserved AAA-ATPases and Peptidase_M41 domains (Fig. S1C), which are known to mediate diverse cellular processes including membrane fusion, proteolysis, and DNA replication.

**Fig. 4:**
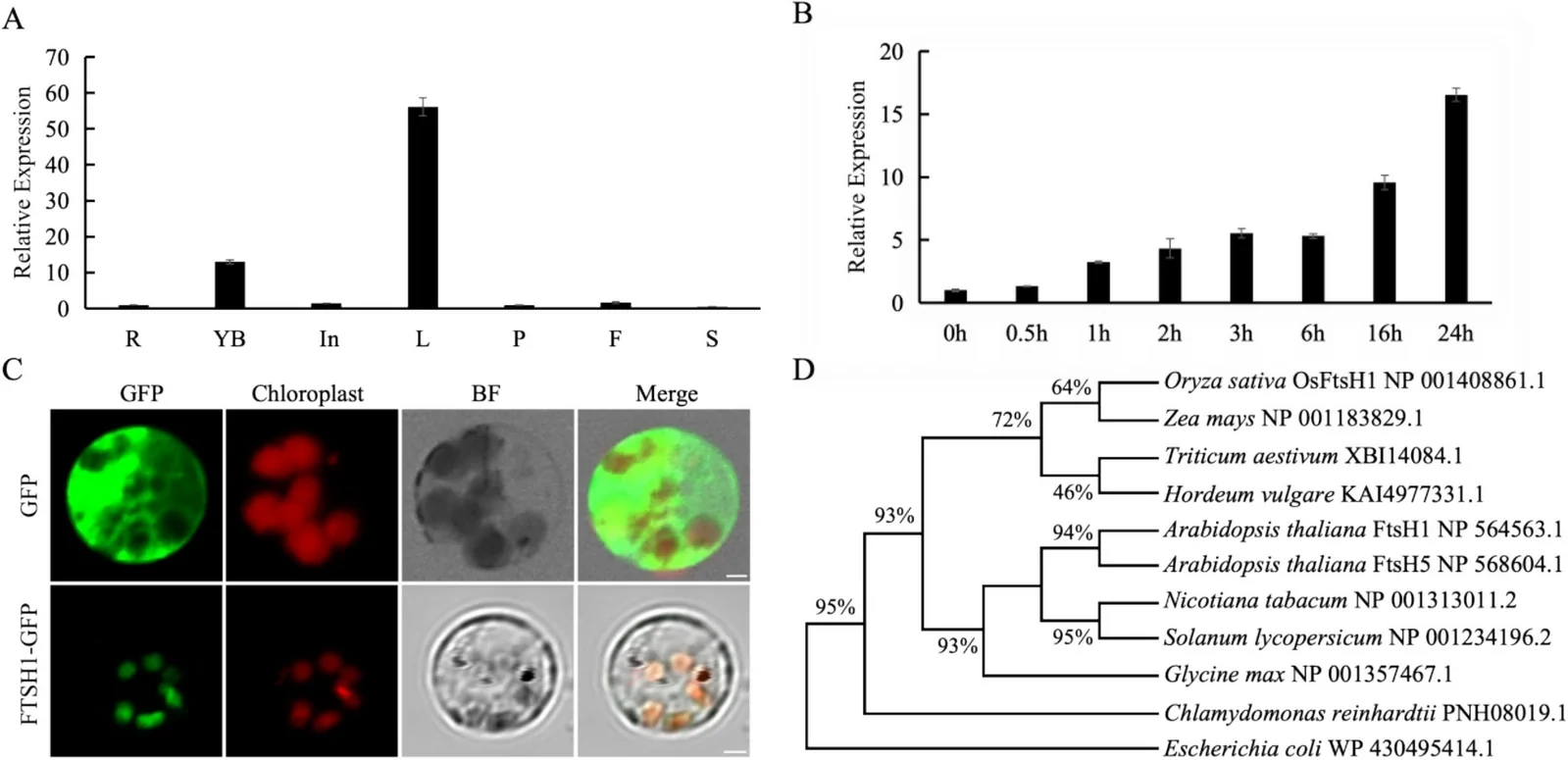
Expression pattern, subcellular localization, and phylogenetic analysis of OsFtsH1. **A** qRT-PCR analysis of *OsFtsH1* expression in different rice tissues. R, root; YB, young bud; In, internode; L, leaf; P, panicle; F, flower; S, seed. **B** Light-induced expression of *OsFtsH1*. Seedlings were exposed to light for 0.5, 1, 2, 3, 6, 16, 24 h. **C** Subcellular localization of OsFtsH1 in rice protoplasts. Green fluorescence represents the GFP signal, while red fluorescence indicates chloroplast autofluorescence. BF, bright field. Bars = 5 μm. **D** Phylogenetic tree constructed based on full-length amino acid sequences of OsFtsH1 homologs in various species. Data in **A**, **B** are presented as mean ± SD (n = 3)

To dissect the evolutionary relationships among FtsH family proteins, we constructed a phylogenetic tree using FtsH orthologs from representative species, including *Oryza sativa*, *Zea mays*, *Arabidopsis thaliana*, *Triticum aestivum*, *Hordeum vulgare*, *Nicotiana tabacum*, *Glycine max*, *Solanum lycopersicum*, *Chlamydomonas reinhardtii*, and *Escherichia coli*. Phylogenetic analysis showed that all FtsH proteins are distinctly divided into two major clades: one clade consists of FtsH proteins from the prokaryote *E. coli* and green alga *C. reinhardtii*, serving as the outgroup; the other clade comprises FtsH proteins from all land plants. The bootstrap value of the node separating C. reinhardtii from all land plant FtsH proteins reaches up to 95%, indicating that FtsH proteins from green algae and land plants share a common ancestral origin yet have undergone substantial sequence divergence during plant terrestrialization, which implies that FtsH family proteins have experienced functional specialization to facilitate plant adaptation to terrestrial habitats, chloroplast development and photosynthetic regulation.Within the land plant clade, monocot species including rice, maize, wheat and barley form a separate subclade, whereas dicot species cluster independently. Among dicots, FtsH proteins of tobacco and tomato exhibit the closest evolutionary relatedness, and Arabidopsis FtsH1 and FtsH5 cluster together with strong bootstrap support. Notably, *Arabidopsis thaliana* encodes a pair of functionally redundant homologous proteins FtsH1 and FtsH5. In contrast, the rice genome encodes only a single FtsH1 homolog without obvious paralogs (Fig. 4D).

Furthermore, multiple sequence alignment demonstrated that the AAA-ATPases and Peptidase_M41 domains of FtsH1 orthologs are highly conserved among rice, maize, *Arabidopsis thaliana*, tobacco, soybean, and tomato (Fig. S4).

### Subcellular Localization and Expression Pattern of *OsFtsH1*

To characterize the spatiotemporal expression pattern of *OsFtsH1* in various organs, we performed quantitative real-time PCR (qRT-PCR) across various rice tissues. *OsFtsH1* transcripts were most abundant in leaves, showed moderate expression in young buds (YB), and were detected at relatively low levels in roots, internodes, panicles, flowers, and seeds (Fig. 4A). Furthermore, when etiolated seedlings grown in darkness were transferred to light conditions, *OsFtsH1* transcript levels increased markedly (Fig. 4B), indicating that its expression is light-inducible.

To define the subcellular localization of OsFtsH1, a fusion construct of *OsFtsH1* with green fluorescent protein (*GFP*) was subjected to transient expression assays in rice protoplasts. The OsFtsH1-GFP fluorescence signal precisely overlapped with chlorophyll autofluorescence, whereas free GFP was diffusely distributed throughout the entire cell (Fig. 4C). These results demonstrate that OsFtsH1 is localized to the chloroplasts, which aligns with its role in regulating chloroplast development.

### Photosynthesis-Related Genes Exhibited Differential Expression Pattern in *osftsh1* Mutant

**Fig. 5:**
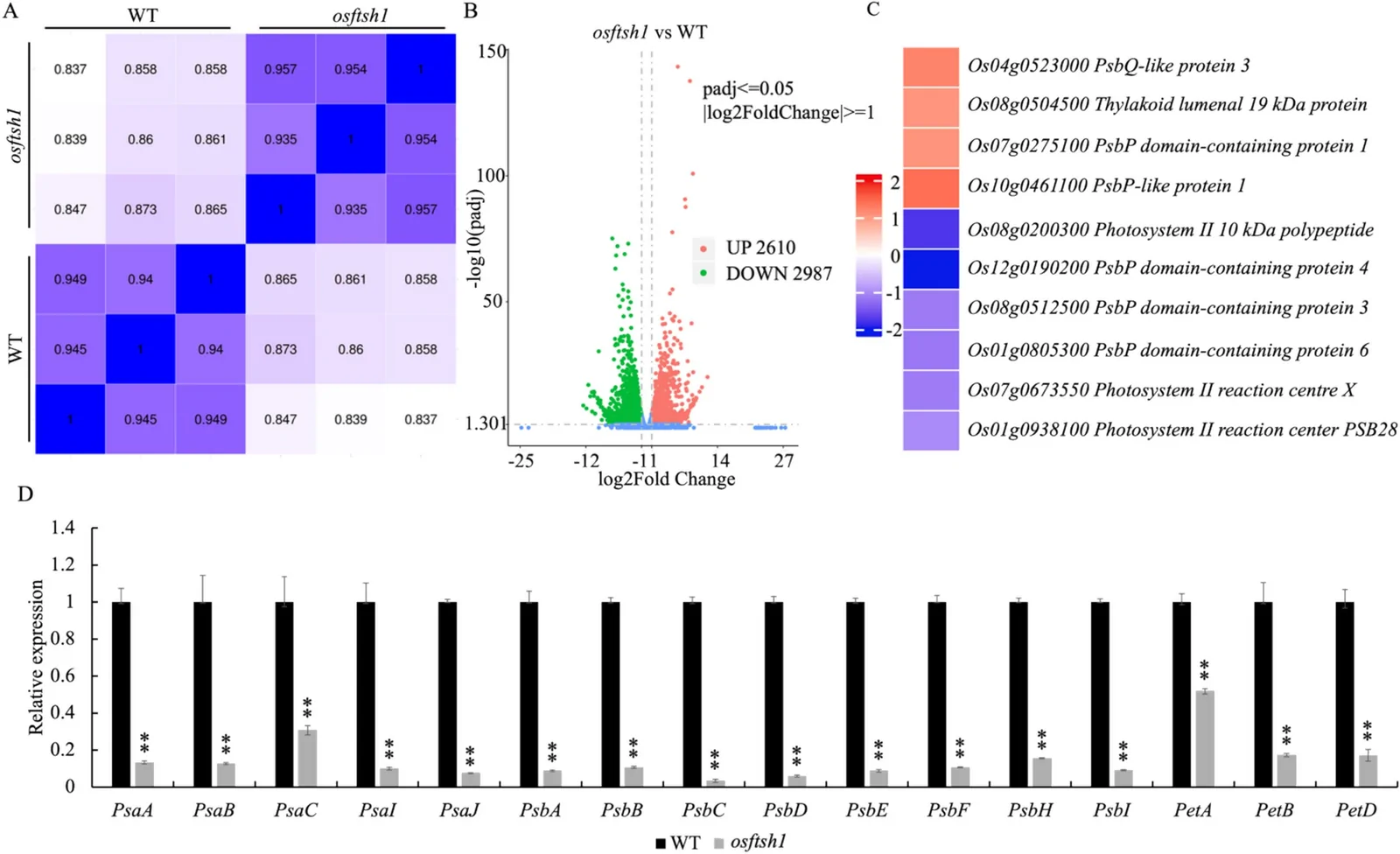
Transcriptome analysis of the WT and *osftsh1* plants. **A** Correlation analysis of gene expression levels between the WT and *osftsh1*. **B** Differentially expressed genes (DEGs) between *osftsh1* and WT plants. **C** Heatmap showing log_2_ fold changes in the expression of nuclear-encoded genes associated with chloroplast development-related genes in WT and *osftsh1* mutants. **D** qRT-PCR validation of plastid-encoded genes in WT and *osftsh1* plants. Data in **D** are presented as mean ± SD (n = 3). Asterisks indicate significant differences based on a two-tailed Student’s t test (**P < 0.01)

To elucidate the biological function of OsFtsH1 in chloroplast development, we performed transcriptome sequencing on WT plants and the *osftsh1* (*wlls1*) mutant. Correlation analysis indicated high similarity among biological replicates and a clear distinction between the two genotypes (Fig. 5A). Compared to the WT, a total of 5597 genes were differentially expressed in the *osftsh1* mutant, of which 2610 were up-regulated and 2987 were down-regulated (Fig. 5B, Table S3). Gene Ontology (GO) enrichment analysis revealed that these differentially expressed genes (DEGs) were significantly enriched in photosynthesis-related terms, consistent with the albino phenotype of *osftsh1* (Fig. S5). Among the 10 photosynthesis-related DEGs (GO:0015979), four nuclear-encoded genes were up-regulated, whereas nine were down-regulated (Fig. 5C). Additionally, quantitative analysis of chloroplast development-related genes revealed that, compared to the WT, the transcription levels of plastid-encoded genes associated with chloroplast development were markedly reduced in the *osftsh1* mutant. These results demonstrate that *OsFtsH1* is essential for the expression of plastid-encoded genes involved in chloroplast development in rice (Fig. 5D).

### OsFtsH1 Sustains Chloroplast Development and Photosynthetic Function by Regulating D1, D2 and CP43 Protein Homeostasis

**Fig. 6:**
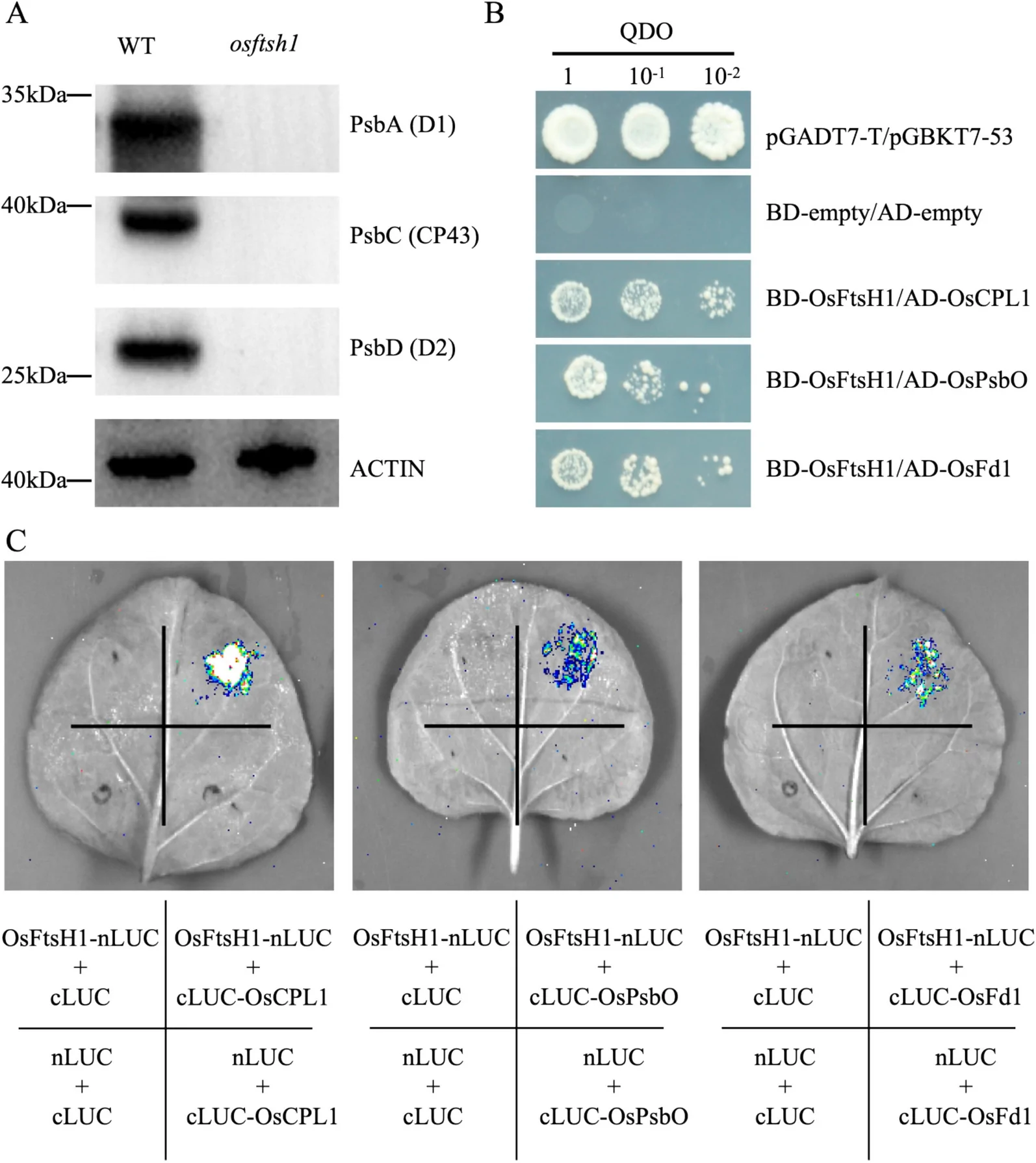
OsFtsH1 sustains the steady-state level of D1, CP43, D2 proteins and interacts with OsCPL1, OsPsbO and OsFd1. **A** The protein levels of D1, CP43 and D2 in WT and *osftsh1* mutants were analyzed by Western blot (WB). **B** Yeast two-hybrid assays confirmed OsFtsH1 physically interacts with OsCPL1, OsPsbO and OsFd1. QDO refers to the quadruple dropout medium (SD/-His-Leu-Trp-Ade). **C** LCI assays in tobacco leaves further verified the physical interactions between OsFtsH1 and OsCPL1, OsPsbO, OsFd1

Chloroplast proteins are vulnerable to damage under environmental stress. Therefore, the maintenance of photosynthetic function relies on a dynamic balance between the rapid degradation of damaged proteins and de novo synthesis to preserve protein homeostasis. D1, D2 and CP43, core subunits of PSII, are particularly dependent on this homeostatic equilibrium under stress conditions. OsFtsH1 contains a conserved domain characteristic of the ATPases Associated with diverse cellular Activities (AAA) proteins, which often possess proteolytic activity. To determine whether the loss of OsFtsH1 function disrupts D1, D2 and CP43 protein homeostasis, we extracted total proteins from WT and *osftsh1* mutant plants, and assessed D1, D2 and CP43 protein abundances via Western blot (WB) analysis. The results demonstrated that D1, D2 and CP43 protein levels were significantly reduced in the *osftsh1* mutant relative to the WT (Fig. 6A), indicating that OsFtsH1 plays an essential role in maintaining D1, D2 and CP43 protein homeostasis.

### OsFtsH1 Interacts with OsCPL1, OsFd1, and OsPsbO to Regulate Chloroplast Development

To systematically decipher the molecular regulatory network of OsFtsH1, we conducted a yeast two-hybrid (Y2H) screen using OsFtsH1 as the bait protein to identify its interacting partners from a rice leaf cDNA library. Sequencing analysis of 48 positive clones identified 41 valid clones, among which 12 clones encoded OsCPL1 (Table S2). This protein acts as a putative regulator of chloroplast signal transduction in rice (Li et al. 2020), and its homolog in Arabidopsis participates in photosynthetic electron transport (Pesaresi et al. 2009); two clones encoded OsPsbO, a core component of the PSII oxygen-evolving complex; and another two clones encoded OsFd1, the major electron carrier that transfers electrons from photosystem I to ferredoxin-NADP^+^ reductase during photosynthetic carbon assimilation. Subsequent pairwise Y2H assays confirmed that OsFtsH1 directly interacts with OsCPL1, OsPsbO, and OsFd1 (Fig. 6B). These results were further verified by a split-luciferase complementation imaging (LCI) assay in Nicotiana benthamiana leaves (Fig. 6C). Collectively, these findings suggest that OsFtsH1 may coordinate chloroplast development and photosynthesis through physical interactions with OsCPL1, OsPsbO, and OsFd1.

### Overexpression of *OsFtsH1* Alters Rice Seedling Responses to IAA and ABA

Transcriptome analysis of the *osftsh1* mutant revealed that differentially expressed genes (DEGs) were significantly enriched in pathways associated with abiotic stress response (Fig. S5). Specifically, within the functional category "response to abiotic stimulus" (GO:0009628), nine DEGs were identified, of which two nuclear-encoded genes were up-regulated and seven were down-regulated (Fig. S6). These findings suggest that OsFtsH1 may exert a crucial regulatory role under various abiotic stress conditions.

**Fig. 7:**
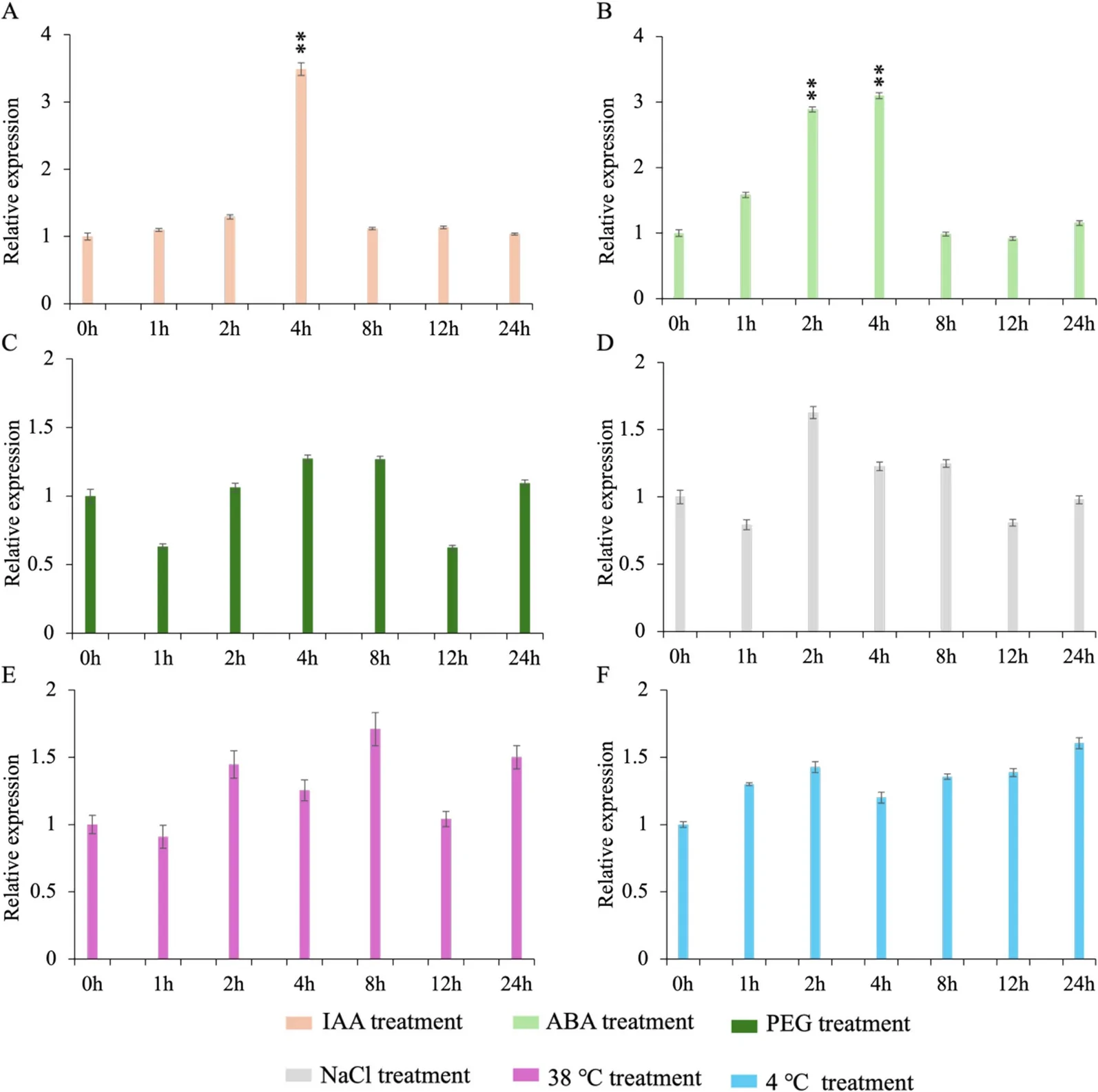
Expression profile of *OsFtsH1* gene under diverse stress conditions. **A** Transcript levels of *OsFtsH1* in response to IAA treatment. **B** Transcript levels of *OsFtsH1* in response to ABA treatment. **C** Transcript levels of *OsFtsH1* under PEG-simulated osmotic stress. **D** Transcript levels of *OsFtsH1* under NaCl-simulated salt stress. **E** Transcript levels of *OsFtsH1* under high-temperature stress (38 °C). **F** Transcript levels of *OsFtsH1* under low-temperature stress (4 °C). WT seedlings were exposed to each treatment, and the transcriptional levels of *OsFtsH1* were measured at 0, 1, 2, 4, 8, 12 and 24 h following treatment. Data are presented as mean ± SD (n = 3). Asterisks indicate significant differences based on a two-tailed Student's *t*-test (**P < 0.01)

To test this hypothesis, we first assessed the effect of various stress treatments on *OsFtsH1* expression. Uniform WT seedlings were treated with six distinct stresses: 20% PEG (osmotic stress), 200 mM NaCl (salt stress), 200 μM IAA, 400 μM ABA, 38 °C heat stress, or 4 °C cold stress. Samples were collected at different time points post-treatment, and *OsFtsH1* transcript levels were quantified by qRT-PCR. The results revealed that *OsFtsH1* expression was significantly altered compared with the WT only under 200 μM IAA and 400 μM ABA treatments (Fig. 7A, B), with no notable differences observed under other stress conditions. These observations indicate that *OsFtsH1* expression is specifically induced by IAA and ABA (Fig. 7C–F). To further clarify the function of OsFtsH1 in abiotic stress response and explore its mechanism, we generated transgenic overexpression lines by introducing the pACT1::*OsFtsH1*-3Flag construct into calli derived from seeds of *osftsh1/OsFtsH1* plants. Four independent transgenic lines (*OsFtsH1*-OE1, OE2, OE3, and OE4) were obtained. Genotypic verification confirmed that these lines were homozygous for the wild-type *OsFtsH1* allele. qRT-PCR results indicated significantly higher transcript accumulation of *OsFtsH1* in these overexpression lines compared with the wild type and non-transgenic controls (NTC1, NTC2) (Fig. S7A). Western blot (WB) assays using an anti-FLAG antibody identified *OsFtsH1*-OE4 as the line with the highest OsFtsH1 protein abundance (Fig. S7B).

**Fig. 8:**
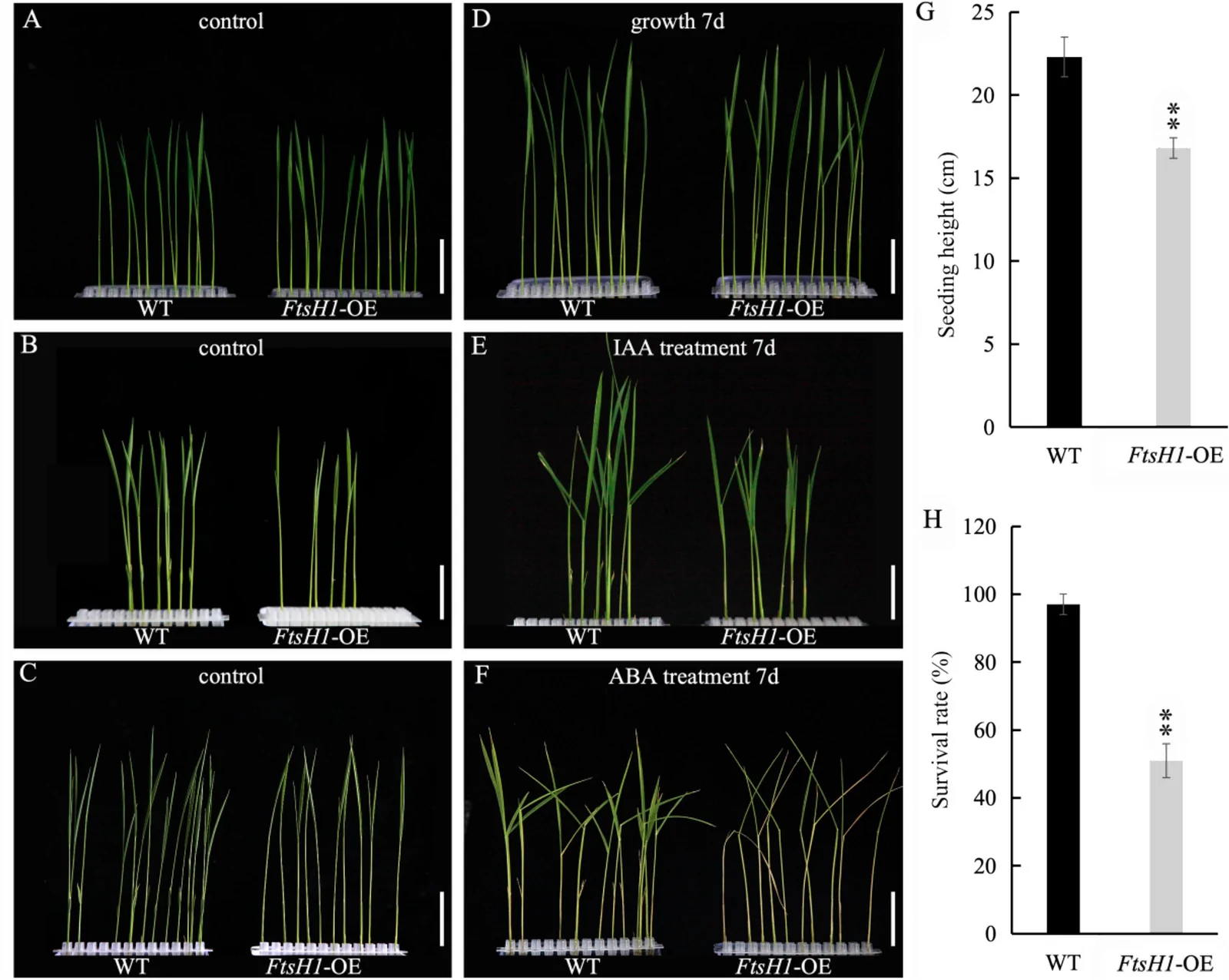
Phenotypic responses of WT and *OsFtsH1*-Overexpressing (*OsFtsH1*-OE) seedlings to IAA and ABA treatments. **A**–**C** Phenotypes of three-leaf-stage WT and *OsFtsH1*-OE seedlings under untreated conditions. **D** Phenotypes of the aforementioned seedlings after 7 additional days of growth without any treatment. **E**, **F** Phenotypes of the seedlings following 7 days of treatment with IAA (**E**) or ABA (**F**). Bars = 5 cm. **G** Quantitative analysis of seedling height after IAA treatment. **H** Survival rate of seedlings after ABA treatment. Error bars represent SD (n = 3, 60 seedlings per replicate). Double asterisks indicate highly significant differences based on a two-tailed Student’s *t*-test (**P < 0.01)

To characterize the role of OsFtsH1 in IAA and ABA signaling, phenotypic analysis was performed on three-leaf-stage *OsFtsH1*-OE4 and WT seedlings treated with 200 μM IAA, 400 μM ABA, or hormone-free nutrient solution for seven days. Under IAA treatment, *OsFtsH1*-OE4 seedlings displayed significantly stronger growth inhibition than the WT (Fig. 8A, B). Under ABA treatment, *OsFtsH1*-OE4 seedlings showed more severe wilting and a drastically reduced survival rate compared with the WT (Fig. 8A, C). In contrast, no significant phenotypic differences were observed between WT and *OsFtsH1*-OE4 plants under control conditions (Fig. 8A). These results indicate that *OsFtsH1* overexpression alters the growth response of rice seedlings to exogenous IAA and ABA application, underscoring its vital regulatory function in hormone-dependent abiotic stress response pathways in rice.

## Discussion

### OsFtsH1 Plays a Vital Role in Chloroplast Development in Rice

The rice (*Oryza sativa*) genome encodes nine FtsH family proteins (Yu et al. 2005; Zhang and Sun 2009), yet the function of FtsH genes in rice remains largely elusive. In this study, we identified a novel chlorophyll-deficient mutant, *wlls1/osftsh1*, which allowed us to clarify the crucial role of the *OsFtsH1* gene in rice chloroplast development. Phylogenetic analysis showed that OsFtsH1 belongs to the type A FtsH protein family that localizes to chloroplasts; it clusters with *Arabidopsis thaliana* FTSH1 and FTSH5, which form a functionally redundant homologous gene pair. Previous studies have established well that the Arabidopsis *ftsh1* single mutant exhibits no obvious phenotypic abnormalities and develops chloroplasts comparable to the WT. By contrast, the *atftsh5* (*var1*) mutant displays only weak leaf variegation and a mildly increased sensitivity to photoinhibition. In contrast, the *atftsh1 atftsh5* double mutant shows an extremely severe phenotype: plants are stunted with albino cotyledons and leaves, fail to form normal chloroplasts, and can survive only heterotrophically on sucrose-supplemented medium. At later developmental stages, these double mutants also exhibit abnormal flowering and complete sterility (Sakamoto et al. 2002; Järvi et al. 2016). These findings indicate that FTSH1 and FTSH5 are functionally redundant for chloroplast biogenesis in Arabidopsis. Given the seedling-lethal phenotype of the rice *osftsh1* single mutant, we infer that OsFtsH1 has a distinct, non-redundant function in rice chloroplast development, and lacks functional compensation from other chloroplast-localized FtsH homologs. Collectively, these results demonstrate that OsFtsH1 plays a crucial role in early chloroplast development in rice. However, this early seedling-lethal phenotype only reflects the essential basal function of OsFtsH1 for seedling survival, while its regulatory functions in photosystem homeostasis, chloroplast development and yield-associated agronomic traits of mature plants remain unknown. To address this limitation, we are currently generating *OsFtsH1* transgenic knockdown lines. Further investigations including phenotypic characterization, physiological measurements (photosynthetic parameters and chloroplast ultrastructure) and validation of molecular interactions will be carried out to dissect the detailed regulatory mechanism of this gene.

### OsFtsH1 is Essential for Sustaining D1, D2 and CP43 Protein Homeostasis

Photosystems, the core functional complexes mediating the light-dependent reactions in photosynthesis, are localized in the thylakoid membrane. Through a tightly orchestrated cascade of energy transfer, photoelectric conversion, and electron transport, they efficiently transduce solar energy into active chemical energy carriers ATP and NADPH. During this process, photosystems harvest light energy via antenna pigment complexes, transfer it to the reaction center to drive charge separation for photoelectric conversion, and then channel high-energy electrons through the photosynthetic electron transport chain. Simultaneously, a transmembrane proton gradient is established to power ATP synthesis. Extensive research has established that chloroplast FtsH proteins are ATP-dependent zinc metalloproteases, which serve as central components of the thylakoid protein quality-control system. A pivotal function of these proteases is to mediate the repair cycle of PSII: they specifically degrade photodamaged and inactivated D1 reaction center protein—a process indispensable for preserving PSII functional stability (Ostersetzer and Adam 1997; Zelisko et al. 2005; Komenda et al. 2006).

In *Arabidopsis thaliana*, loss-of-function mutants of *atftsh1* (*var1*) and *atftsh2* (*var2*) exhibit reduced accumulation of photosystem I (PSI) complex proteins, whereas the abundance of the PSII core protein D1 remains comparable to that of the wild type. Moreover, no significant differences are observed in the accumulation of psaA/B transcripts or their translation efficiency between *var1*/*var2* mutants and the wild type controls, suggesting that the impairment of PsaA/B protein accumulation in these mutants occurs at post-translational stages (Järvi et al. 2016). In tobacco (*Nicotiana tabacum*), NtFtsH1 and NtFtsH2 represent the major isoforms constituting the chloroplastic FtsH complex, as demonstrated by their concurrent down-regulation in FtsH RNA interference (RNAi) lines. These transgenic tobacco lines display a photoinhibition-sensitive phenotype, characterized by high-light-induced suppression of PSII activity and delayed degradation of the D1 protein (Mirkovic et al. 2017).

Notably, the D1 protein was completely undetectable in the *osftsh1* mutant (Fig. 6A). This starkly contrasts with the Arabidopsis *var1*/*var2* mutants, which show reduced PSI proteins but unaltered D1 abundance (Järvi et al. 2016), as well as tobacco FtsH RNAi lines, which exhibit retarded D1 degradation (Kato et al. 2012). The complete absence of detectable D1 in the *osftsh1* mutant strongly suggests that loss of OsFtsH1 function triggers a feedback regulatory mechanism that directly disrupts the intrinsic synthesis-degradation equilibrium of the D1 protein. We hypothesize that this mechanism either represses key regulators governing D1 synthesis or activates additional robust thylakoid protein degradation pathways independent of FtsH proteases. Ultimately, these perturbations preclude D1 from reaching even basal accumulation levels. This "complete blockage of D1 accumulation" in rice may represent a unique adaptive strategy to counteract specific environmental stresses or to sustain the overall efficiency of the photosynthetic apparatus.

### The OsFtsH1-OsFD1 Interaction Modulates Photosynthetic Carbon Metabolism

Photosynthesis consists of two interconnected phases: the light-dependent reactions centered on PSII and PSI, and the carbon-fixing Calvin-Benson cycle. Specifically, electrons generated by photoexcitation in PSII are transferred in a sequential manner through the cytochrome b₆f complex, PSI complex, ferredoxin (Fd), and ferredoxin-NADP⁺ reductase (FNR) (Andersen et al. 1992; Raines 2003; Gollan et al. 2015; Caspy and Nelson 2018). In rice (*Oryza sativa* L.), loss-of-function *osfd1* mutants exhibit a chlorotic phenotype and are seedling-lethal starting from the three-leaf stage. These mutants exhibit markedly reduced contents of chlorophyll a, chlorophyll b, and carotenoids. Their chloroplast structure is abnormal, characterized by sparse grana lamellae, absence of starch granules, and an increase in osmiophilic granules, and chloroplast number is decreased. Concurrently, transcript levels of key Calvin cycle genes (e.g., TK, Prkase, SBPase) and the activities of key enzymes (e.g., PGK, GADPH, FBPase) are significantly down-regulated. These perturbations collectively lead to impaired accumulation of photosynthetic products such as glucose, starch, and soluble sugars. Given the central role of OsFD1 in mediating photosynthetic electron transport, yeast two-hybrid assays in this study confirmed that OsFtsH1 physically interacts with OsFD1 at the protein level. This interaction suggests that OsFtsH1 may fine-tune OsFD1-dependent electron transport processes, thereby facilitating the balanced supply of ATP and NADPH, sustaining the operational efficiency of the Calvin-Benson cycle, and ensuring the steady accumulation of carbohydrates and other photosynthetic products. Furthermore, this finding reveals a novel regulatory node for elucidating the molecular regulatory network underlying plant adaptive responses to light conditions.

### OsFtsH1 Mediates the Abiotic Stress Response in Rice

Accumulating evidence has established that FtsH family proteins are inducible by diverse abiotic stresses and serve as core regulators in orchestrating plant stress adaptation (Chen et al. 2018). For instance, the transcription of *AtFtsH6* and *AtFtsH11* in *Arabidopsis thaliana* (Sedaghatmehr et al. 2016; Chen et al. 2018), FtsH in wheat (Rangan et al. 2020), and NEEDLE1 (an FtsH family member) in maize is significantly upregulated under high-temperature stress (Liu et al. 2019). Similarly, AtFtsH2/8 in Arabidopsis (Wang et al. 2015) and FtsH8 in soybean are co-induced by concurrent drought and high-temperature stress (Das et al. 2016), while AtFtsH1/5, AtFtsH6, and AtFtsH8 are synergistically activated under high-light and high-temperature conditions (Balfagón et al. 2019). Functional characterization further demonstrates that loss-of-function mutations in AtFtsH11 drastically enhance Arabidopsis sensitivity to high temperatures (Chen et al. 2018), whereas heterologous overexpression of TsFtsH8 from *Thellungiella salsuginea* effectively enhances plant cold tolerance (Liu et al. 2016). Additionally, the core chloroplast-localized FtsH proteins in Arabidopsis (AtFtsH1/5, AtFtsH2/8, AtFtsH6, AtFtsH7/9) alleviate high-light-induced photoinhibition of the photosynthetic apparatus by mediating the repair of photodamaged D1 protein in PSII (Wagner et al. 2011; Yoshioka-Nishimura et al. 2014; Kato et al. 2015; Krynická et al. 2015), highlighting the crucial role of the FtsH family in plant abiotic stress responses. In the present study, RNA-seq analysis of the *osftsh1* mutant revealed that differentially expressed genes (DEGs) were significantly enriched in pathways related to "response to abiotic stimulus", suggesting that rice OsFtsH1 may exert critical regulatory functions under multiple stress conditions. Subsequent abiotic stress treatment assays showed that *OsFtsH1* expression was specifically and markedly induced by IAA and ABA. Moreover, under exogenous IAA and ABA treatments, *OsFtsH1* overexpression lines exhibited substantially altered stress sensitivity compared with the WT. Collectively, these results confirm that OsFtsH1 plays a core role in rice abiotic stress response and provide a pivotal target for dissecting the molecular mechanism underlying plant stress resistance regulation.

## Materials and Methods

### Plant Materials and Growth Conditions

The *wlls1* mutant was derived from the japonica rice variety Yandao 8 (Y8), which was subjected to ^60^Co γ-ray irradiation. In the present study, Y8 was used as the wild-type (WT) control. Plants were cultivated in a controlled-environment incubator under a 14-h light (20,000 lx)/10-h dark photoperiod.

### Measurement of Pigment Contents

Pigment content was quantified following a previously described method with minor modifications (Zhang et al. 2021). Briefly, fresh leaf samples (0.1 g) harvested from WT and *wlls1* mutant plants at the three-leaf stage of the M4 generation were cut into small pieces and immersed in 10 mL of a 1:1 (v/v) ethanol:acetone mixture for extraction. After incubation in darkness for 12 h, the absorbance of each extract was measured at 470, 649 and 665 nm using a microplate reader spectrophotometer to quantify carotenoids, chlorophyll b and chlorophyll a, respectively. The pigment concentrations were calculated according to standard equations based on the recorded absorbance values. All experiments were performed with three biological replicates.

### Transmission Electron Microscopy Analysis

Fresh leaf samples from the fully expanded second true leaves at the three-leaf stage of the WT and *wlls1* mutant plants were cut into small pieces and fixed in 3% glutaraldehyde prepared in 0.1 M phosphate buffer. After being washed three times with the same buffer, the samples were post-fixed in 1% OsO_4_ for 1.5 h. Following fixation, the samples were washed again three times with 0.1 M phosphate buffer. The fixed samples were dehydrated through a graded acetone series (50, 70, 90, and 100% acetone; 15 min per step, with 20 min for the final step). Subsequently, the dehydrated samples were infiltrated and embedded in Epon 812 resin using the following steps: first, incubation in an acetone-resin mixture (2:1, v/v) for 0.5 h; second, immersion in an acetone-resin mixture (1:2, v/v) at 37℃ for 1.5 h; and finally, incubation in pure Epon 812 resin at 37 ℃ for 3 h. The resin-embedded samples were polymerized through sequential incubation at 37, 45, and 60 ℃, with each temperature stage maintained for 24 h.

Ultrathin sections (70 nm thick) were prepared using an LKB-V ultramicrotome, and then double-stained with uranyl acetate and lead citrate for 15 min each, and observed under a JEOL-1200E transmission electron microscope. Images were captured using a MORADA-G2 digital camera system.

### Map-Based Cloning of the Target Gene

For map-based cloning of *WLLS1*, heterozygous *wlls1/WLLS1* plants were crossed with the indica rice variety Xieqingzao to construct a mapping population. Recessive individuals from the F_2_ and F_3_ generations were screened to identify recombinants. Based on the sequence polymorphisms between Xieqingzao and Y8, InDel markers were developed using publicly available data from the National Center for Biotechnology Information (NCBI) at http://www.ncbi.nlm.nih.gov. Using these markers, fine-mapping progressively delimited the mutation to a narrow genomic interval on chromosome 6. All polymorphic markers used for mapping are listed in Supplementary Table S1.

### Gene Editing, Complementation and Overexpression Assay

The CRISPR/Cas9-mediated gene editing approach was performed as previously described (Ma et al. 2015). The target sequence was designed using the online tool CRISPR-GE (http://skl.scau.edu.cn/). The OsU6a promoter, target sequence, and guide RNA (gRNA) scaffold were assembled via a two-step PCR strategy. In the first round of PCR, specific primers were used to link the target sequence to the OsU6a promoter and gRNA scaffold. The resulting overlapping PCR products were then mixed and used as templates in a second round of PCR to fuse all three elements. The final PCR products were digested with restriction enzyme *Bsa*I and inserted into the pYLCRISPR/Cas9-MH vector. After sequence verification, the recombinant construct was introduced into *Agrobacterium tumefaciens* strain EHA105 and used to transform calli derived from Yandao 8. Mutants were identified by genotyping transgenic plants.

For the complementation and overexpression assays, the coding sequence (CDS) of *OsFtsH1* was cloned into the pCAMBIA23A vector, with its expression driven by the rice *Actin1* promoter. The resulting construct was introduced into the calli derived from seeds of *wlls1/WLL1* plants using the same Agrobacterium-mediated transformation procedure as employed for gene editing. Complemented transgenic lines were confirmed by detecting the presence of the *OsFtsH1* transgene and analyzing the restoration of the wild-type phenotype in the *wlls1* mutant background. By contrast, overexpression lines were identified through transgene verification and phenotypic characterization in the homozygous *WLL1*/*WLL1* wild-type background.

### qRT-PCR Assay

Total RNA was extracted from rice tissues using TRIzol reagent. Reverse transcription was performed using HiScript III RT SuperMix for qRT-PCR (Vazyme), which includes a gDNA removal reagent (gDNA wiper) to eliminate genomic DNA contamination. Quantitative PCR (qPCR) analysis was conducted using ChamQ SYBR qPCR Master Mix (Vazyme) on a Bio-Rad CFX96 real-time PCR instrument. The PCR amplification protocol consisted of 40 cycles, with each cycle including denaturation at 95 ℃ for 10 s and annealing/extension at 60 ℃ for 30 s. All reactions were performed in triplicate, with the ubiquitin gene used as an internal control. The primers used for qRT-PCR are listed in Supplementary Table S1.

### Subcellular Localization of OsFtsH1

The coding sequence (CDS) of *OsFtsH1* was amplified and cloned into the pJIT163-GFP vector, in-frame with the green fluorescent protein (GFP) coding sequence. The resulting plasmids and an empty pJIT163-GFP control were independently transfected into rice protoplasts via PEG-mediated transformation. Fluorescence signals were captured 24 h post-transformation using a confocal laser scanning microscope (Carl Zeiss LSM 710, Oberkochen, Germany). GFP and red fluorescence signals (chloroplast autofluorescence) were excited at 488 nm and 561 nm, respectively, and their emissions were collected at 500–540 nm for GFP, and 600–650 nm for red fluorescence. The primers used for amplifying the *OsFtsH1* CDS are listed in Supplementary Table S1.

### RNA-seq Analysis

Total RNA was isolated from leaves of 14-day-old WT and *osftsh1* plants using reagents from Invitrogen Life Technologies. For library construction, 2 µg of total RNA was subjected to ribosomal RNA depletion using the Epicentre Ribo-Zero™ rRNA Removal Kit. First-strand cDNA synthesis was carried out using random primers, followed by RNA strand degradation with RNase H. The resulting cDNA fragments were size-selected (400–500 bp) and purified using the AMPure XP system (Beckman Coulter, Beverly, CA, USA). The purified libraries were sequenced on an Illumina NovaSeq 6000 platform to generate data for downstream analysis.

### Yeast Two-Hybrid (Y2H) Assays

The CDS of *OsFtsH1* was cloned into the pGBKT7 vector (bait vector), and the recombinant plasmid (pGBKT7-OsFtsH1) was transformed into the yeast strain Y2Hgold. The self-activation activity of the resulting construct was tested by observing yeast growth on SD-Ade-His-Trp dropout medium. A commercially available cDNA library (provided by Nanjing Ruiyuan Biotechnology Co., Ltd.) was screened using the Y2H system to identify proteins interacting with OsFtsH1. Positive interacting clones were confirmed by Sanger sequencing. Subsequently, the full-length CDS of each candidate interacting protein was subcloned into the pGADT7 vector (prey vector). The recombinant prey plasmid and pGBKT7-OsFtsH1 were co-transformed into Y2Hgold yeast. Protein–protein interactions were verified by checking yeast growth on stringent quadruple dropout medium (SD-Leu-Trp-His-Ade).

### Luciferase Complementation Imaging (LCI) Assay

The CDSs of rice *OsFtsH1*, *OsCPL1*, *OsPsbO* and *OsFd1* were fused to the nLUC and cLUC fragments in the pCAMBIA1300-nLUC and pCAMBIA1300-cLUC vectors, respectively. *Agrobacterium tumefaciens* strains harboring the respective constructs were co-infiltrated in pairwise combinations into the same leaves of *Nicotiana benthamiana* and cultured at 28 °C for 48 h. Following application of 0.2 mM luciferin, luminescence signals were captured using a low-light cooled CCD imaging system.

### Protein Extraction and Western Blot Analysis

Leaves from two-leaf-stage WT and *osftsh1* mutant seedlings were harvested, flash-frozen in liquid nitrogen, and ground to a fine powder. Total protein was extracted using a Plant Total Protein Extraction Kit (PTE011-5T). Proteins were separated by sodium dodecyl sulfate–polyacrylamide gel electrophoresis (SDS-PAGE) and transferred onto a nitrocellulose membrane via wet transfer. The membrane was blocked for 1 h at room temperature with Tris-Buffered Saline with Tween-20 (TBST) containing 1% bovine serum albumin (BSA) and 5% skim milk, followed by incubation with primary antibodies overnight at 4 °C. After washing with TBST, the membrane was incubated with horseradish peroxidase-conjugated secondary antibodies for 1 h at room temperature. Target proteins were detected using an enhanced chemiluminescence (ECL) system (Liu et al. 2024).

### Stress Treatment

Uniformly germinated seeds of Y8 (WT) and *OsFtsH1*-overexpressing (*OsFtsH1*-OE) were selected and cultured in half-strength Murashige and Skoog (1/2MS) liquid medium under controlled environmental conditions (14 h light/10 h dark, 28 °C) until the three-leaf stage. For stress treatments, WT seedlings at three-leaf stage were subjected to six distinct stress conditions: osmotic stress (20% PEG-6000), salt stress (200 mM NaCl), 200 μM indole-3-acetic acid (IAA), 400 μM abscisic acid (ABA), high temperature (38 °C), or low temperature (4 °C). Each treatment contained 60 seedlings. Seedlings were sampled at 0 (control, before treatment), 1, 2, 4, 8, 12, and 24 h post-treatment for subsequent qRT-PCR analysis. For phenotypic comparison under hormone stress, three-leaf stage seedlings of WT and *OsFtsH1*-OE were treated with either 200 μM IAA or 400 μM ABA, while untreated seedlings served as controls. Phenotypes were observed and recorded after 7 days of treatment.

### Statistical Analysis

Statistical significance was assessed using Student’s *t* test, with a *P*-value < 0.05 considered statistically significant. All data are presented as the mean ± standard deviation (SD) from three independent biological replicates.

## Supplementary Information

Supplementary Material 1.

Supplementary Material 2.

## Data Availability

All relevant data can be found within the manuscript and its supporting materials.
