# Supplementary material for: A FtsH-Like Zinc Metalloprotease, OsFtsH1, Coordinates Chloroplast Development and Photosystem II Homeostasis in Rice
Source: Rice (N Y). 2026 Jul 11;19:66. doi: 10.1186/s12284-026-00936-2 (PMC13642237; doi:10.1186/s12284-026-00936-2)
Supplement: Supplementary file 1 [file 12284_2026_936_MOESM1_ESM.docx]

**Figure S1 Map-based cloning of the candidate gene** ***WLLS1*.** (A) The number of recombinant plants is indicated below the corresponding molecular markers. The red arrowhead denotes the mutation site of *WLLS1* in the *wlls1* mutant; blue arrowheads indicate the CRISPR/Cas9 target sites in the *osftsh1-cr* mutant line. (B) Genomic location and size of the deletion within *WLLS1* in the *wlls1* mutant. (C) Schematic diagram of the AAA and Peptidase_M41 domains in the OsFtsH1 protein.

**Figure S2 Amino acid sequences of OsFtsH1 in *wlls1* and *osftsh1-cr* mutants.** Red arrows indicate the position of frameshift mutation in the amino acid sequence.

**Figure S3 Relative expression levels of *OsFtsH1* in *wlls1* and *osftsh1-cr* mutants as** **determined by qRT-PCR.** Data are presented as mean ± SD (n = 3). Asterisks indicate significant differences compared with WT based on a two-tailed Student’s *t*-test (***P* < 0.01).

**Figure S4** **M****ultiple sequence alignment of orthologous OsFtsH1 proteins.** The blue line indicates the AAA-ATPase domain, and the pink line indicates the Peptidase_M41 domain. **Amino acid residues highlighted in red denote conserved sites across the aligned sequences.** *Os*, *Oryza sativa*; *Zm*, *Zea mays*; *At*, *Arabidopsis thaliana*; *Nt*, *Nicotiana tabacum*; *Gm*, *Glycine max*; *Sl*, *Solanum lycopersicum*.

**F****igure S5** **GO enrichment analysis of DEGs from RNA-seq data of leaves from WT and *osftsh1* plants.** BP = Biological Process, CC = Cellular Component, MF = Molecular Function.

**Figure S****6** **Analysis of abiotic stress-responsive genes in WT and *osftsh1* seedlings.** The heatmap displays log₂ fold changes in the expression of genes related to abiotic stress response between the WT and *osftsh1* mutant plants.

**Figure S7** **Expression levels of *OsFtsH1* in WT and overexpression lines.** (A) Relative expression levels of *OsFtsH1* in overexpression lines *OsFtsH1*-OE1, 2, 3 and 4 were quantified by qRT-PCR. NTC (non-transgenic control) refers to WT plants. Data are shown as mean ± SD (n = 3). ** indicates highly significant differences based on Student’s *t* test (***P* < 0.01). (B) Protein levels of OsFtsH1 in selected overexpression lines, *OsFtsH1*-OE1, 2, 4 were detected by Western blot (WB). ACTIN served as the loading control for normalization.

| **Table S1. Primers used in this study (5’-3’).** | | |
| --- | --- | --- |
| Assay | Name | Sequence |
|  | M1-F | ACGGATGAAAATTAATGGAA |
|  | M1-R | AAAAATATAGGGATGCAAAA |
|  | M2-F | AGGAATCTGTGTTTAGTGTC |
| Mapping | M2-R | TTGATCGAGGGATTGATTAC |
|  | M3-F | AATCGGTCCACTCATGTAAT |
|  | M3-R | TATTAATGCGAGTGCTTGTG |
|  | M4-F | TAGGTATGAGCTCAGGAAGC |
|  | M4-R | ACCCGCTACTCTTCTCTTCT |
|  | M5-F | CCAGCTAATCCTAACAGCAT |
|  | M5-R | ACCTAAGCTCCTCAAACACA |
|  | M6-F | CCATACTGTCCAAATACCGT |
|  | M6-R | TGCCTAAAATGTCAAAACCT |
|  | M7-F | TCATGTAGATTGTTACATAC |
|  | M7-R | AGTAATAAACTAAGTCTTAA |
|  | M8-F | CTCTGTTTTTCGATTGAACC |
|  | M8-R | AGGTAGGAGAAAGGCAAGAC |
|  | M9-F | GCATCTCCATTTCTCAATTT |
|  | M9-R | TTCGTGGATAGCTAGGAAAA |
|  | M10-F | TATTAGCTAGAGGGTGGTGC |
|  | M10-R | ATTTCAAGACAAAGGCAAAA |
|  | M11-F | CAAACCTGCTGTATCCTACC |
|  | M11-R | TAGGCATGTGTTCACAAAAA |
|  | M12-F | TTGTGTTGTTGTTGGCTTTA |
|  | M12-R | TAACTACTCCCAGCCATTTC |
| Overexpression | OsFtsH1-OE-F | TaggtagaagaggtacccgggATGGCGCCGCCGTGCTCCATCTC |
|  | OsFtsH1-OE-R | atccttgtaatctccgtcgacAGCGACAAACAACTCTGCCTGAC |
| qRT-PCR | Ubiquitin-F | CAAGATGATCTGCCGCAAATGC |
|  | Ubiquitin-R | TTAACCAGTCCATGAACCCG |
|  | OsFtsH1-RT-F | TCTCCTCCGCCTCCCACCTCCT |
|  | OsFtsH1-RT-R | CGCGGGCACGTCGGCGAGGGCG |
|  | PsaA-RT-F | TTCCCAGGAGTCTTGCTTCG |
|  | PsaA-RT-R | TGGTGGTACTGGAACTTCGC |
|  | PsaB-RT-F | GGGCCCACTAAGCATGAAGG |
|  | PsaB-RT-R | TGTTGCTCCTTGCTCTTCAGAT |
|  | PsaC-RT-F | TGATTGAGGAGGCACTTGACA |
|  | PsaC-RT-R | CGCTATATCTTGCTGGTGCAG |
|  | PsaI-RT-F | GCCAACAACAGTGTGAAATCCA |
|  | PsaI-RT-R | TTTGTTGGCGCAGTTTGGAG |
|  | PsaJ-RT-F | GGAGGATCTGAACTCCCTGC |
|  | PsaJ-RT-R | CTGCTGGTGCACCCTAAAAG |
|  | PsbA-RT-F | TGTGGCCGCTCATGGTTATT |
|  | PsbA-RT-R | CCCTACTACAGGCCAAGCAG |
|  | PsbB-RT-F | GCATCTGCTGCACTGGAAAC |
|  | PsbB-RT-R | TCGCCAAGAAGATTTCTCTGAGT |
|  | PsbC-RT-F | GGACCACTCAGCGTCAAAGA |
|  | PsbC-RT-R | GACGCTCCTTTCTGCGAAGT |
|  | PsbD-RT-F | ACCCGAAACAGCTGTCCAAA |
|  | PsbD-RT-R | CCAGCAAATTTCTTTGAGTCCGT |
|  | PsbE-RT-F | GCCAAAGCATGACCAGAACT |
|  | PsbE-RT-R | TGAGGTACTCATTGCGGCTG |
|  | PsbF-RT-F | GCATGCTTGTGGCTCAACTT |
|  | PsbF-RT-R | CAACCAGGTGCTTGTTGGTG |
|  | PsbH-RT-F | CACGCTGAGCCATCTCAAGG |
|  | PsbH-RT-R | ATACTTCCTGTGGCGAACCT |
|  | PsbI-RT-F | AGCGCCGGAATAAACAAGACA |
|  | PsbI-RT-R | GAGCGTTGGAGACATTCAAGC |
|  | PetA-RT-F | GCACGGAAGAACGAATTGCT |
|  | PetA-RT-R | TTTTGCACGTACTCGCTCGT |
|  | PetB-RT-F | TTTTCACCAAGCACGCACTG |
|  | PetB-RT-R | ACACTTGCTGCTGACTGGTT |
|  | PetD-RT-F | ACACTTTCCGCACATCCACT |
|  | PetD-RT-R | CGCTGCATAAAGCCCTTCCT |
| cas9 | OsFtsH1-cas9-F | GTACTAGAGGCCCCCTCCCgttttagagctagaaat |
|  | OsFtsH1-cas9-R | GGGAGGGGGCCTCTAGTACCggcagccaagccagca |
| Subcellular localization | OsFtsH1-GFP-F | ggacagggtacccggggatccATGGCGCCGCCGTGCTCCATCTC |
|  | OsFtsH1-GFP-R | agtgtcgactctagaggatccAGCGACAAACAACTCTGCCTGAC |
| Y2H | OsFtsH1-BD-F | aggccgaattcccggggatcATGGCGCCGCCGTGCTCCATCT |
|  | OsFtsH1-BD-R | ccgctgcaggtcgacggatccTTAAGCGACAAACAACTCTGCC |
|  | Fd1-AD-F | gccatggaggccagtgaattcATGGCGGCGACGGCACTG |
|  | Fd1-AD-R | cagctcgagctcgatggatccTTAGATGAGGTCGTCCTCCTTGTG |
|  | CPL1-AD-F | gccatggaggccagtgaattcATGGCCGCTCTTTCCTCTG |
|  | CPL1-AD-R | cagctcgagctcgatggatccTTAGTTGACGGTGACCTTGCC |
|  | PsbO-AD-F | gccatggaggccagtgaattcATGGCAGCATCGCTCCAA |
|  | PsbO-AD-R | cagctcgagctcgatggatccCTACTCGAGCTGCGCGTACC |
| LCI | OsFtsH1-JW771-F | acgggggacgagctcggtaccATGGCGCCGCCGTGCTCCATCT |
|  | OsFtsH1-JW771-R | cgcgtacgagatctggtcgacTTAAGCGACAAACAACTCTGCC |
|  | Fd1-JW772-F | tacgcgtcccggggcggtaccATGGCGGCGACGGCACTG |
|  | Fd1-JW772-R | acgaaagctctgcaggtcgacTTAGATGAGGTCGTCCTCCTTGTG |
|  | CPL1-JW772-F | tacgcgtcccggggcggtaccATGGCCGCTCTTTCCTCTG |
|  | CPL1-JW772-R | acgaaagctctgcaggtcgacTTAGTTGACGGTGACCTTGCC |
|  | PsbO-JW772-F | tacgcgtcccggggcggtaccATGGCAGCATCGCTCCAA |
|  | PsbO-JW772-R | acgaaagctctgcaggtcgacCTACTCGAGCTGCGCGTACC |

**Table S2 Complete list of positive clones identified via yeast two-hybrid screening using OsFtsH1 as the bait.**
